# Intramuscular injection of vectorized-scFvMC1 reduces pathological tau in two different tau transgenic models

**DOI:** 10.1186/s40478-020-01003-7

**Published:** 2020-08-06

**Authors:** Francesca Vitale, Jasmin Ortolan, Bruce T. Volpe, Philippe Marambaud, Luca Giliberto, Cristina d’Abramo

**Affiliations:** 1 Institute of Molecular Medicine, The Litwin-Zucker Center for Alzheimer’s Disease & Memory Disorder, The Feintein Institutes for Medical Research, Manhasset, NY USA; 2 Donald and Barbara Zucker School of Medicine at Hofstra/Northwell, Hempstead, NY USA; 3 Institute of Molecular Medicine, Center for Autoimmune and Musculoskeletal Disease, The Feinstein Institutes for Medical Research, Manhasset, USA; 4 Northwell Health Neuroscience Institute, Northwell Health System, Manhasset, NY USA

**Keywords:** Vectorized antibodies, AAV, scFv, Tau, Immunotherapy, Intramuscular injection

## Abstract

With evidence supporting the prion-like spreading of extracellular tau as a mechanism for the initiation and progression of Alzheimer’s disease (AD), immunotherapy has emerged as a potential disease-modifying strategy to target tau. Many studies have proven effective to clear pathological tau species in animal models of AD, and several clinical trials using conventional immunotherapy with anti-tau native antibodies are currently active. We have previously generated a vectorized scFv derived from the conformation-dependent anti-tau antibody MC1, scFvMC1, and demonstrated that its intracranial injection was able to prevent tau pathology in adult tau mice. Here, we show that, in a prevention paradigm and in two different tau transgenic models (JNPL3 and P301S), a one-time intramuscular injection of AAV1-scFvMC1 generated a long-lasting peripheral source of anti-tau scFvMC1 and significantly reduced insoluble and soluble tau species in the brain. Moreover, our data showed that scFvMC1 was internalized by the microglia, in the absence of overt inflammation. This study demonstrates the efficacy of intramuscular delivery of vectorized scFv to target tau, and suggests a new potential application to treat AD and the other tauopathies.

## Introduction

The microtubule-associated protein tau plays a physiological role in microtubule stabilization, axonal growth and cytoskeletal dynamics in neurons, but its aggregation characterizes several neurological diseases classified as tauopathies, including Alzheimer’s disease (AD) [1–5]. As increasing evidence supports the existence of tau as an extracellular protein and the concept of its trans-cellular propagation as a mechanism for the initiation and progression of AD, tau has become an attractive target for immunotherapy in animal models of AD and related tauopathies [6–13]. A conspicuous amount of data has been produced by different laboratories, including ours, showing reduction of tau pathology in transgenic animal models using tau monoclonal antibodies, with a different degree of success according to which epitopes were targeted [14–26]. However, passive immunotherapy using conventional antibodies in humans presents several potential limitations such as low permeability to cross the blood-brain barrier (BBB), detrimental inflammatory reactions and microhemorrhages associated to the treatment, requirement for repeated dosing, patients compliance and high costs [27–30]. To overcome these issues, several groups have engineered antibodies as fragments, i.e. single chain variable fragment (scFv), to be used in combination with gene delivery strategies based on the use of viral vectors: vectorized anti-Aß (amyloid-beta) and anti-tau scFvs have previously shown benefits in models of AD [31–45]. ScFvs consist of the smallest functional antigen-binding domain of an antibody (Ab) exhibiting comparable antigen-binding affinity as the parent immunoglobulin, reduced size, improved pharmacokinetic in terms of tissue penetration and lack of an Fc receptor-mediated inflammatory response [46–48]. Due to their short systemic half-life in vivo [49, 50], in order to reach a sustained and long-lasting expression scFv are generally cloned in adeno associated viral vectors (AAVs) and delivered by one-time injections [34, 46, 47]. We have previously shown [43] that the AAV vectorized-scFvMC1, a recombinant version of the native anti-tau conformational mAb (monoclonal antibody) MC1, significantly reduces brain pathological tau in adult JNPL3 mice, by one-time intracranial injection. In parallel with AAV-based delivery, Spencer et al. [44] have shown that systemic injection of a lentiviral vector (LV) carrying a scFv directed to 3Rtau and enhanced for brain penetration (LV-3RT-apoB) was able to reduce tau accumulation, neurodegeneration and behavioral deficit in a tau transgenic model. Although using third-generation lentiviral vectors has emerged as a promising therapeutic option for conditions as primary immunodeficiencies and cancers, it is still necessary to understand the long-term safety and efficacy of these vectors in humans, especially in respect to their potential for insertional oncogenesis [51]. In this context, using AAVs is considered safer than LVs.

Direct delivery of AAV into the brain has been tested in a number of clinical trials [52–57] and parallel efforts have been done over the past years to develop new brain-targeted AAVs to treat CNS diseases in vivo using a systemic delivery approach [58]. In this respect, peripheral administration provides obvious advantages to AAV gene therapy: a non-invasive route of injection, lack of surgery-related side effects, improved patient compliance and costs. In this study we propose to develop a novel therapeutic approach for AD and tauopathies by using intramuscular (IM) delivery of AAV-vectorized scFv. To date, there has been little effort to develop peripheral protocols targeting skeletal muscle to tackle CNS disorders. IM injection provides a quick, easy, non-invasive and safe route of administration, and can routinely be performed in virtually any setting. Also, skeletal muscles are an ideal target tissue for AAV transduction because individual fibers are large, multinucleated and with minimal cellular turnover [59]. Taking advantage of the practical features and biological properties of this delivery approach, the aim of the present study was to generate a long-lasting peripheral niche able to produce and release anti-tau scFv in the circulation to target cerebral tau, without transducing vital organs such as liver, kidneys and heart. A similar strategy has been previously tested in a mouse model of AD, where intramuscular delivery of AAV1 vectored anti-Aß scFv was able to reduce Aß load in brain [35, 45]. Similarly, gene therapy for Alpha-1 antitrypsin (AAT) deficiency has been developed in humans using recombinant AAV1 serotype, demonstrating a continued stable transgene expression at 5 years after transduction [60, 61].

In this study, for the first time, we demonstrate the in vivo feasibility and efficacy of targeting pathological tau in the brain, by employing intramuscular delivery of vectorized anti-tau scFv. Two different tau transgenic models, the JNPL3 and P301S mice, received a single IM injection of AAV1-scFvMC1, showing a significant reduction of tau pathology, with some differences between strains. Moreover, no signs of inflammation were observed upon AAV1-scFvMC1 immunization, showing that tau clearance does not involve Fc-receptor-mediated and microglia-associated detrimental inflammatory response. However, our in vitro and in vivo data point to the microglia as a player in the uptake and clearance of scFv-tau, despite the absence of Fc, adding to the potential mechanisms of action of tau immunotherapy.

In summary, our data support the peripheral intramuscular route as an effective, feasible and safe delivery approach for AAV-scFv-based anti tau immunotherapy, with relevant translational potential applications to other tauopathies and brain disorders.

## Methods

### ScFv-MC1 design and sub-cloning into AAV1

The light and heavy-chain variable domains corresponding to the MC1 antibody were sequenced employing the MCLAB antibody service (San Francisco, CA). As previously published [43] the V_H_ and V_L_ chains were joined together by a 15 amino acid residues linker (Gly_4_Ser)_3_. 5′-terminal signal peptide (SP) and 3′-terminal Myc and His6X tags were added. The AAV packaging and purification service was provided by Vector Biolab (Malvern, PA). ScFv-MC1 was sub-cloned into the adeno-associated viral vector serotype 1 (AAV1) under the control of the synthetic strong CAG (CMV-chicken beta actin-rabbit beta globin) promoter. In order to enhance expression of the transgene, the WPRE Woodchuck hepatitis virus (WPRE) post-transcriptional regulatory element was added 5′ of the Myc and His6X tags.

### Tau transgenic mice

JNPL3 mice obtained from Taconic (Germantown, NY) express 0N4R human tau with the P301L mutation that causes frontotemporal dementia in humans, under the mouse prion promoter. JNPL3 mice develop NFTs-like pathology as early as 4.5 months and in later stages progressive deterioration of the motor function [62]. Homozygous P301S were obtained from Dr. Michel Goedert (Cambridge, UK) [63]: these mice, on pure C57BL/6 background, express 0N4R human tau carrying the P301S mutation, under the control of the neuron-specific murine Thy-1 promoter, and they develop widespread tau pathology affecting cerebral cortex, hippocampus and brain stem as early as 6 months, and partial paralysis of the lower limbs by 8 months of age. Animals were treated according to the current regulations for the proper handling of research animals, following an approved IACUC protocol.

### ScFvMC1 purification

ScFvMC1 purification was performed as previously published [43]. Briefly, scFv-MC1 was cloned into the mammalian expression vector pcDNA3.1 (Genewiz, South Plainfield, NJ) and transfected into HEK293T, using Lipofectamine 2000 (Invitrogen, Carlsbad, CA). After 48 h of transfection, the scFv released into the conditioned medium was affinity purified using a Ni-Sepharose High Performance column (GE Healthcare, Port Washington, NY). The efficiency of purification was tested using an immunosorbent assay employed to assess the antigen–binding specificity of the scFvMC1, as previously described [43]. Starting material, flow through and eluted fractions were tested to check for proper enrichment of the purified material. The purified scFv-MC1 was checked on Coomassie-stained SDS-PAGE gel for proper molecular weight.

### Infrared conjugation and intravenous (IV) injections

ScFv-MC1 and MC1 have been conjugated with IRDye 800CW, using IRDye 800CW protein Labeling Kit Low-MW or High-MW respectively (LI-COR Biosciences, Lincoln, NE), according to the manufacturer instructions. Briefly, scFv-MC1 and MC1 were dialyzed in 50 mM potassium phosphate buffer pH 8.5 at 4 °C, overnight; the pH was then adjusted with 1 M potassium phosphate buffer to 9. After 2 h incubation, the unconjugated dye was removed using desalting spin columns (Zeba Desalt Spin Columns, Thermo Scientific). ScFvMC1 and MC1 antigen-binding reactivities were measured against an MC1 specific peptide [43] by immunosorbent assay, in order to exclude loss of activity upon conjugation.

To verify the ability of scFvMC1 and MC1 to cross the blood brain barrier, IV injection was performed in 3-month-old JNPL3 mice using 100 μg of the antibodies: saline, scFvMC1-IRDye, MC1-IRDye, or unlabeled antibodies were injected (*n* = 3 per group). Mice were anesthetized with isoflurane and the injections performed retro-orbitally. Mice were sacrificed 2 h post injection; brains were harvested and dissected into cortex (Ctx), hindbrain (HB) and hippocampus (Hip). Homogenization was performed in 1X RIPA buffer (Thermo Fisher Scientific, Waltham, MA) with the Mini protease inhibitor cocktail (Roche, Indianapolis, IN). Brain samples from each region were spotted on 0.45 μm nitrocellulose followed by IR signal acquisition at 789 nm, using Sapphire Biomolecular Imager (Azure Biosystems, Dublin, CA).

### Intra-muscular (IM) injections

AAV1-CAG-scFvMC1 or AAV1-CAG-eGFP were injected at a dose of 2X10^11^ GC per mouse. Each AAV was diluted in PBS at a final volume of 50 μl, and a one-time intramuscular injection was administrated in the right tibialis. Injections were performed upon anesthesia with isoflurane.

Twenty-six females JNPL3 (n  =  13 per group) mice were injected at 3 month of age and sacrificed 4 months later. The P301S line was injected at 2 month of age and sacrificed 4 months later; we used twelve females P301S in total (*n* = 6 per group). Overall, 26 JNPL3 and 12 P301S mice were employed in this study.

### Brain extracts and tissues preparation

Mice were sacrificed by isoflurane overdose, decapitated and processed as described previously [14]. The brain was removed and divided at the midline so that just one half of the brain was dissected for biochemical analysis. Cortex, hippocampus and hindbrain were homogenized separately using an appropriate volume of homogenizing buffer, a solution of Tris-buffered saline (TBS), pH 7.4, containing 10 mM NaF, 1 mM Na_3_VO_4_ and 2 mM EGTA, plus the complete Mini protease inhibitor cocktail (Roche). Supernatants were analyzed for protein concentration using DC Protein Assay (Bio-Rad Laboratories, Hercules, CA). Brain homogenates were stored at − 80 °C and used for separate measurement of soluble and insoluble tau. Soluble tau was measured as heat-stable preparation (hsp) from brain. Hsp were prepared by adding 5% ß-Mercaptoethanol and 200 mM NaCl to the brain homogenates. Samples were then heated at 100 °C for 10 min and cooled at 4 °C for 30 min. After centrifuging at 14,000 g in a table-top microcentrifuge at 4 °C for 15 min, supernatants were collected and 5X sample buffer (Tris-buffered saline, pH 6.8 containing 4% SDS, 2% ß-Mercaptoethanol, 40% glycerol and 0.1% bromophenol blue) was added. To obtain insoluble tau preparations (INS), homogenates were thawed and spun at 14,000 g for 10 min at 4 °C. The collected supernatants were centrifuged at 200,000 g for 30 min at 4 °C; the pellets were then re-suspended in homogenizing buffer and centrifuged again at 200,000 g for 30 min at 4 °C. The final pellets were re-suspended in 1X sample buffer and heated at 100 °C for 10 min to efficiently dissociate the insoluble tau fraction.

Liver, kidney and heart were harvest and homogenated using 1X RIPA buffer with the complete Mini protease inhibitor cocktail (Roche). Protein concentration were analyzed using DC Protein Assay (Bio-Rad Laboratories) and samples were prepared for western blotting. For tibialis and gluteus maximum muscles protein were extracted in skeletal muscle homogenizing buffer (20 mM Tris, 137 mM NaCl, 2.7 mM KCl, 1 mM MgCl2, 1% Triton X-100, 10% glycerol, 1 mM EDTA and 1 mM dithiothreitol) plus the complete Mini protease inhibitor cocktail (Roche). Tissue were mince using a Douncehomogenizer, sonicated and then let vortexed overnight at 4 °C. The supernatant, containing the protein extract, was collected after 15 min centrifugation at 14,000 g and used in immunoblot, as described later.

### Tau ELISA

Levels of total and phosphorylated tau were assessed using the Low-tau ELISA (enzyme-linked immunosorbent assay) protocol previously published [64, 65]. 96-well plates were coated for 48 h at 4 °C with specific purified monoclonal tau antibodies (DA31, CP13, PHF1, RZ3) at a concentration of 6 μg/ml. After washing, plates were blocked for 1 h at RT using StartingBlock buffer (Thermo Fisher Scientific). Brain samples and standards were diluted in 20% SuperBlock buffer (Thermo Fisher Scientific) in 1XTBS and loaded on the plates. Once the samples were added, the total tau detection antibody DA9-HRP, diluted 1:50 in 20% SuperBlock in 1XTBS, was added to the samples and tapped to combine. Plates were then incubated overnight at 4 °C. Next day, 1-Step ULTRA TMB-ELISA (Thermo Fisher Scientific) was added for 30 min at RT, followed by 2 N H_2_SO_4_ to stop the reaction. Plates were read with Infinite m200 plate reader (Tecan, San Jose, CA) at 450 nm.

### Immunoblotting

An aliquot of the total lysates was used for western blotting (WB). 0.1% SDS was added to the lysates, followed by sonication (3 cycles, 10 s each). Samples were run on 4–20% Criterion Tris-HCl gels (Bio-Rad Laboratories) and electrophoretically transferred to a nitrocellulose membrane (Thermo Fisher Scientific). Residual protein-binding sites were blocked by incubation with 5% non-fat milk in 1XTBST (1X TBS plus 0.1% Tween 20) 1 h at RT, followed by an overnight (O/N) incubation at 4 °C with primary antibodies diluted in 20% SuperBlock buffer (Thermo Fisher Scientific) in 1XTBST. Mouse anti-tubulin (Thermo Fisher Scientific) were diluted 1:5000; anti-Myc-tag 9B11 (Cell Signaling, Danvers, MA) was diluted 1:1000. Appropriate isotypes secondary antibodies HRP-conjugated were diluted 1:2000 or 1:10000 in 5% non-fat milk 1XTBST, and added for 1 h at RT. Every step was followed by 3 or 4 washes in 1X TBST. Detection was performed using Pierce ECL Western Blotting Substrate (Thermo Fisher Scientific) or SuperSignal West Dura extended duration substrate (Thermo Fisher Scientific) and exposed to x-ray films.

### Immunocytochemistry, immunofluorescence and image analysis

Tau staining and immunofluorescence were performed according to standardized protocols [14, 43]. After decapitation, half of the brain was fixed overnight in 4% paraformaldehyde at 4 °C. Serial sections were cut from the fixed brain half on a vibratome, conserved in TBS (50 mM Tris, 150 mM NaCl, pH 7.6)/0.01% NaN_3_, and stained on 24-well plates with a panel of tau antibodies. Endogenous peroxidases were quenched with 3% H_2_O_2_/0.25% Triton X-100/1XTBS for 30 min. Non-specific binding was blocked with 5% non-fat milk-1XTBS for 1 h at RT. Primary antibodies were used as follows: anti tau antibodies RZ3 and MC1 (1:500), CP13 and PHF1 (1:5000); all antibodies were diluted in 5% non-fat milk-1XTBS, and incubated O/N at 4 °C, shaking. Biotin-conjugated secondary antibodies (SouthernBiotech, Birmingham, AL) directed against the specific isotypes were diluted 1:1000 in 20% SuperBlock, left for 2 h at RT, and lately Streptavidin-HRP (SouthernBiotech) was incubated for 1 h. Staining was visualized by 3,3′-Diaminobenzidine (Sigma-Aldrich, St. Louis, MO). Images were acquired using Olympus BH-2 bright field microscope (Waltham, MA); analyzed and processed using ImageJ/Fiji software (NIH). Semi-quantification was done on the hippocampal quadrant CA1 and on the entorhinal cortex by using the measure particles tool, working with 8-bit images and adjusting the threshold.

For immunofluorescence, sections were pre-incubated 5 min at RT in 1XTBS (Gibco, Carlsbad, CA) containing 0.2% TritonX100 (Sigma-Aldrich). After blocking 1 h at RT with a solution containing 5% normal goat serum (Sigma-Aldrich) diluted in 1XTBS/0.2% Triton, sections were incubated with primary antibodies diluted in 1% normal goat serum in 1XTBS/0.2% Triton: anti-Myc-Alexa Flour555 1:500 (EMD Millipore), Iba-1 1:1000 (Wako Chemicals, Richmond, VA), anti-CD68 1:200 (Bio-Rad Laboratories) and RZ3 (anti-tau pThr231) 1:500. After washing 3X in 1XTBS/0.2% TritonX100, Alexa Fluor secondary antibodies − 488 and − 568 and − 350 (Invitrogen) were added at 1:1000 or 1:2000 dilutions for 1 h at RT, in different combinations in order to obtain multiple labeling images. DAPI (Invitrogen) was used to counterstain. Brains slices were then mounted on slides and let dry 20 min before being coverslipped using Vectashield hard set anti-fade mounting (Vector Laboratories, Burlingame, CA). Sections incubated without primary antibody were used as negative controls. Images were acquired using Zeiss 880 confocal laser microscope (Peabody, MA). Integrated intensity was quantified using NIH ImageJ (NIH) on raw images, with background fluorescence subtraction on pre-defined ROIs.

For Iba-1 VIP-substrate staining (Vector Laboratories, Burlingame, CA), antigen retrieval was performed using 1X Dako Target Retrieval solution (Agilent Dako, Santa Clara, CA, USA) in distilled water/0.5% Triton, at 95–99 °C for 5 min. After washing, endogenous peroxidases were quenched with 3% H_2_O_2_/0.25% Triton X-100/1XTBS for 30 min. Sections were incubated in 5% normal goat serum (Sigma Aldrich) in 1XTBS/0.1% Triton. Primary polyclonal antibody, anti Iba-1 (Wako Chemicals, Richmond, VA), was diluted 1:2000 in 1% normal goat serum in 1XTBS/0.1% Triton and let incubate O/N at 4 °C. Biotin-conjugated goat anti-rabbit secondary antibody (SouthernBiotech) were used at 1:2000 in 20% SuperBlock (ThermoFisher) in 1X TBS/0.05% Triton, left for 2 h at RT, and lately Streptavidin-HRP (SouthernBiotech) was incubated for 1 h. Staining was visualized using Vector VIP Substrate (Vector Laboratories) following the manufacture’s specifications. After washing with distilled water slides were mounted and coverslipped. Microglia were imaged on AxioImager Z1 microscope (Zeiss) at 63x oil and 0.58 μm z-steps to capture 3 ROIs across the stratum radiatum of the CA1 subfield of the hippocampus for each animal (5 mice per group, 10 cells per mouse imaged: 50 cells per treatment group analysed). The microglia process morphology was categorized with a score from 0 to 3, following the criteria described by Schafer et al. [66–68]: 0 (> 15 thin processes with multiple branches), 1 (5–15 thick processes with branches), 2 (1–5 thick processes with few branches), 3 (no clear processes). All analyses were performed in blind.

On peripheral organs, histology was performed by HistoWiz Inc. (histowiz.com) using a Standard Operating Procedure and fully automated workflow. Samples were processed, embedded in paraffin, and sectioned at 4 μm. Immunohistochemistry was performed on a Bond Rx autostainer (Leica Biosystems) with enzyme treatment (1:1000) using standard protocols. Slides were stained with hematoxylin and eosin and anti-NFkb. Bond Polymer Refine Detection (Leica Biosystems) was used according to manufacturer’s protocol. After staining, sections were dehydrated and film coverslipped using a TissueTek-Prisma and Coverslipper (Sakura). Whole slide scanning (40X) was performed on an Aperio AT2 (Leica Biosystems).

### Primary mouse microglia cultures and uptake experiment

Cultures were prepared from post-natal C57BL/6 mouse pups at 2 days of age. Whole brains were trypsin digested and made into a cell suspension. Cells were seeded in flasks pre-coated with 0.1 mg/ml poly-D-lysine (Sigma-Aldrich) and maintained in DMEM supplemented with 10% heat-inactivated FBS (Gibco) and 1% Pen-Strep (Gibco). Medium was supplemented with 5 ng/ml Macrophage Colony Stimulating factor (M-CSF) (Thermo Fisher Scientific) diluted in PBS supplemented with 0.1% sterile filtered BSA (Sigma-Aldrich). At DIV10 microglia were isolated by orbital shaking at 150 RPM for 1 h and the supernatant was seeded in 12-well plates with 300,000 cells per well. Experiments were performed on the subsequent day. PHF-tau (paired helical filaments) [69] was added to microglia at a concentration of 1 μg/ml as determined by total tau ELISA. ScFvMC1 was added at a concentration of 10 μg/ml. To allow for immune complex formation, PHF-tau and scFvMC1 were mixed in medium and pre-incubated at 37 °C for 30–45 min prior to addition to cells. Mixing was performed two times during incubation by repeated manual pipetting. The 2 h incubation was performed in medium without serum. All experiments were performed in triplicate, with each treatment group in quadruplicate. The amount of PHF-tau in medium at the end of the experiments was assessed using the same low-tau ELISA previously described.

### Stereotaxic intracranial injection

Intra-hippocampal injections of AAV vectors were performed according to a stereotaxic surgery protocol previously published [43]. Briefly, under sterile conditions, 3-month-old P301S mice were anesthetized and secured on a stereotaxic frame (David Kopf instruments, Tujunga, CA). Mice received bilateral hippocampal injection of AAV preparations using a neuro syringe with a 33 gauge needle (Hamilton, Reno, NV), using the following coordinates: AP − 2.1 from bregma, ML +/− 2.0 from bregma, DV − 1.8 below dura. Animals were treated according to the current regulations for the proper handling of research animals, following an approved IACUC protocol.

### Flow cytometry on adult mice microglia

Microglia was isolated from 6-month-old P301S mice treated with AAV5-scFv-MC1 and AAV5-null injected mice. In this experiment, we used an AAV-null construct instead of AAV-eGFP, since our goal was to ascertain the uptake of the scFv by microglia and since eGFP may interfere with flow cytometry analysis. Mice were anesthetized and cold PBS-perfused. After dissection, the forebrain was minced with a Dounce homogenizer in ice cold HBSS, filtered onto 70 μm cell strainer, and centrifuged at 300 g for 5 min at 4 °C. Tissue was dissociated using Neural Dissociation Kit P (MACS Miltenyi Biotec, Auburn CA) according to the manufacturer’s instruction. Myelin debris were removed using Myelin Removal Beads II (MACS Miltenyi Biotec). Briefly, after neural dissociation, samples were spun at 300 g for 10 min at 4 °C and incubated 15 min with Myelin Removal Beads in 0.5% BSA in 1X PBS. Cells suspension was then loaded onto a pre-washed MACS LS column and placed in the magnetic field of MACS Separator. The magnetically labeled myelin was retained within the column while unlabeled cells run through [70]. Cells suspension, myelin depleted, was then washed twice with FACS buffer (0.05% BSA, 0.02% sodium azide in 1X PBS) and stained with Live/Dead-Pacific Blue (Thermo Fisher Scientific). Surface staining was performed using CD11b-PE and CD45-APC/Cy7 antibodies (BD Biosciences, Franklin Lakes, NJ) in order to select microglia from other monocytes. After fixation and permeabilization with BD Cytofix/Cytoperm Fixation/Permeabilization Solution Kit (BD Biosciences) cells were stained with anti-Myc Tag Antibody AlexaFluor-647 (Thermo Fisher Scientific). Debris, doublets and dead cells were excluded using fsc/ssc, fsc-h/fsc-w and Pacific Blue gates, respectively. BD CompBeads (BD Biosciences) were used for calibration of flow cytometer. Samples were analyzed on a BD LSRFortessa and data processed using FlowJo software (Treestar).

### Tau and anti-scFvMC1 antibodies detection in serum

A detailed protocol was previously published to detect total tau in serum [71]. Upon sacrifice mice were bled, samples collected and allowed to clot for 30 min at RT. After cooling for 15 min, samples were spun at 14,000 g for 10 min at 4 °C; supernatants were collected and then re-spun at 14,000 g for 5 min at 4 °C. The final supernatants correspond to the serum samples. In order to detect tau in serum, samples were diluted 1:3 in 0.2 M NaOAc, pH 5.0 and heated at 90 °C for 15 min. After the heat treatment, samples were allowed to cool at 4 °C for 15 min, and then spun at 15,000 g for 10 min. Supernatants were collected and 1 M Tris buffer was added to neutralize the pH. After diluting 1:2 in 20% Superblock, samples were loaded on the total tau ELISA (DA31 capture).

In order to detect antibodies directed to the scFvMC1, 96-well plates were coated with purified scFvMC1 at 6 μg/ml for at least 24 h. Plates were washed 3X and blocked for 1 h using StartingBlock (Thermo Fisher Scientific). Plates were washed 5X and 50 μl of sera added in triplicate at 1:1000 dilution in 20% SuperBlock (Thermo Fisher Scientific). After 1 h incubation plates were washed 5X and 50 μl of goat anti-mouse non-specific IgG HRP-conjugated (SoutherBiotech, Birmingha, AL) was added and incubated for 1 h. Finally, Bio-Rad HRP Substrate Kit has been used for the detection and plates were read with Infinite m200 plate reader (Tecan) at 415 nm.

### Statistical analysis

Quantitative data were analyzed using the dedicated software GraphPad Prism V.6 (GraphPad software Inc., CA). Unpaired t test with Welch’s correction was performed when the parametric assumption of normality (D’Agostino-Pearson omnibus test) was accomplished. When not, non parametric Mann-Whitney test was performed instead. Statistical significance was set at *P* < 0.05. Error bars represent the standard error of the mean (SEM).

## Results

### ScFvMC1 is detected in the brain homogenates upon intravenous injection of the purified scFv

**Fig. 1:**
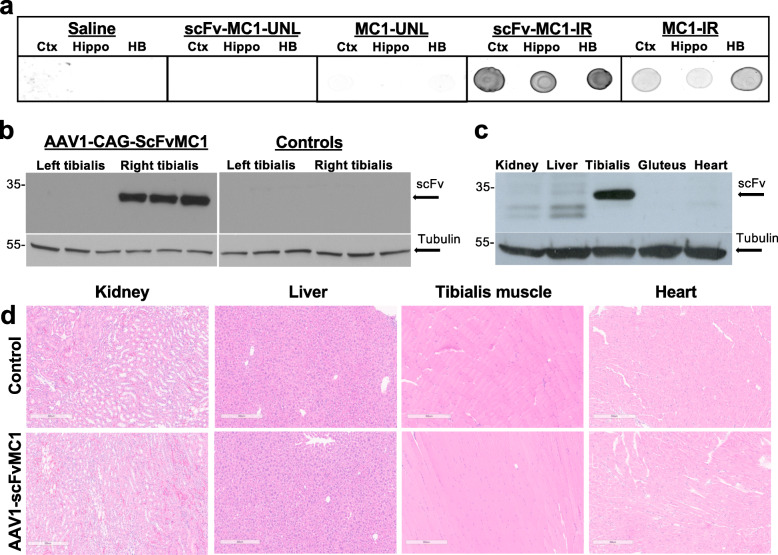
ScFvMC1 detection and peripheral organs’ morphology. **a** Six-month old mice (JNPL3 shown) were IV-injected with saline, 100 μg of IRDye-labelled antibodies (scFvMC1-IR and MC1-IR) and unlabeled antibodies (scFvMC1-UNL and MC1-UNL). Two hours post-injection, brain homogenates from cortex (Ctx), hippocampus (Hip) and hindbrain (HB) were spotted on nitrocellulose and absorbance of the IRDye label measured. Saline and unlabeled antibodies showed no signal (Sapphire Biomolecular Imager, Azure Biosystems). **b**, **c** AAV1-CAG-scFvMC1 was injected in tibialis muscle of P301S or JNPL3 (JNPL3 shown), mice sacrificed 4 months later and peripheral organs harvested. Tissue lysates were analyzed for scFvMC1 expression with anti-Myc/tag antibody showing localized expression in the injected site (**b**, right tibialis, Controls = AAV1-eGFP) while other peripheral organs did not show scFv signal (**c**). ScFvMC1: MW around 30 kDa; tubulin is used as housekeeper: MW at 55 kDa. **d** Hematoxylin and eosin (H&E) staining was performed on kidney, liver, tibialis muscle and heart: representative images of each organs show no significant changes in morphology (Controls = AAV1-CAG-eGFP; Bright field microscope, scale bar: 300 μm)

We first asked whether scFvMC1 crosses the BBB and targets the brain [72, 73]. After intra-peritoneal (IP) peripheral injection, we were not able to detect brain scFvMC1 using antibodies directed against Myc or 6-His (tags present on our scFv construct), via IHC, WB or ELISA (not shown). We have thus performed a proof-of concept experiment, testing BBB penetration via retro-orbital IV injection of 100 μg of purified scFvMC1, either unlabeled (UNL) or infrared-conjugated (IR), in adult JNPL3 and P301S mice; a careful comparison was performed between scFvs and native MC1 variants, including saline as a negative control. Given the scFv’s short half-life, mice were sacrificed 2 h post-injection and perfused with ice-cold PBS/Heparin. The serum concentration of both scFvMC1-IR and the native MC1-IR were calculated in the amount of 50 ng/μl. At termination of the experiment, scFvMC1 was detected in cortex, HB and hippocampus (Fig. 1a): its amount was in the range of 0.1–0.2% of the serum concentration, in line with the current literature [40, 72, 74, 75], confirming the feasibility of a peripheral approach that relies on a sustained release of scFv in the circulation.

### AAV1-scFvMC1 specifically transduces muscle cells upon one-time intramuscular injection

Since scFvMC1 efficiently crosses the BBB, we have next focused on selecting a peripheral tissue to be transduced by the AAV-scFv construct, to generate a stable source of antibody, with the goal to circumvent vital organs. Skeletal muscle is considered an ideal target tissue for AAV transduction because individual fibers are large, multinucleated and with minimal cellular turnover [35, 59, 76]. Hence, we have selected the AAV1 serotype to produce a stable muscular niche able to continuously produce anti-tau scFvMC1 and release it into the circulation to target cerebral tau. In a prevention protocol, given the different timeline in the development of tau pathology in the two mice models selected, 3-month-old JNPL3 and 2-month-old P301S mice were injected in the right tibialis anterior muscles with AAV1-CAG-scFvMC1 and sacrificed 4 months post-injection, at 7 or 6 months of age respectively. To ascertain transduction of our AAV system, transgene expression in the injected site was assessed by immunoblotting. Figure 1b shows a specific band around 30–35 kDa corresponding to scFv-MC1 expression in the injected tibialis. Other peripheral organs failed to show expression of scFv-MC1 (Fig. 1c), confirming targeted local delivery and the high specificity of the AAV1-CAG system. No premature death or body weight loss were reported throughout the study. Moreover, no changes in tissues morphology at the time of sacrifice were detected as assessed by hematoxylin and eosin (H&E) (Fig. 1d); the inflammatory state, also unchanged, was assessed using NF-kB staining (Supplementary Fig. 1).

### AAV1-scFvMC1 intramuscular injection significantly decreases the insoluble tau burden in both P301S and JNPL3 mice

**Fig. 2:**
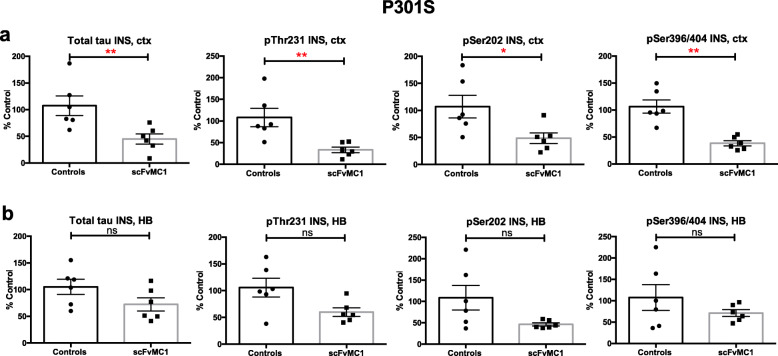
Insoluble tau levels are significantly reduced in P301S brains. **a** In cortex, insoluble tau levels (INS) were significantly decreased in the scFvMC1 treated group (*n* = 6*)* compared to the control cohort (n = 6) (*p < 0.05; **p < 0.01; non parametric Mann-Whitney test). **b** In HB, insoluble tau showed a trend of reduction in the scFvMC1 group (n = 6) compared to the controls (n = 6): total tau (*p* = 0.0931), pThr231(*p* = 0.0931), pSer202 (*p* = 0.1320), pSer396/404 (*p* = 0.5887). Graphs are expressed as % Control, and means +/− SEM

**Fig. 3:**
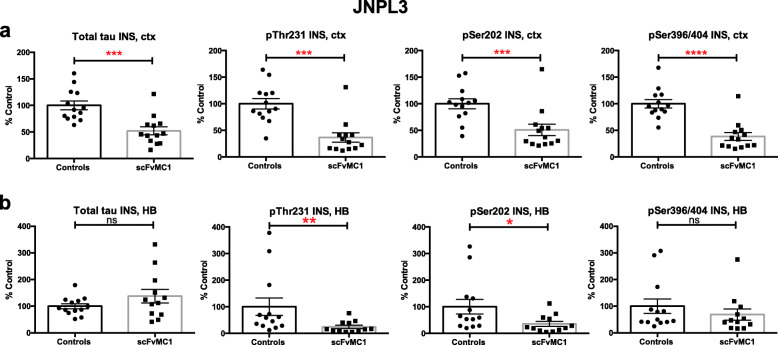
Insoluble tau levels are significantly reduced in JNPL3 brains. **a** In the JNPL3 mice, insoluble tau (INS) was significantly reduced in cortex (ctx) in the AAV1-CAG-scFvMC1 group (scFvMC1) (*n* = 13) compared to the AAV1-CAG-eGFP injected cohort (Controls) (n = 13). Both total and phosphorylated tau (three different epitopes pThr231, pSer202 and pSer396/404) were significantly reduced (***p < 0.001; ****p < 0.0001; non parametric Mann-Whitney test). **b** Total and pSer396/404 insoluble tau were unchanged in the JNPL3 hindbrain (HB) in the treated scFvMC1 mice (*n* = 13) compared to the controls (n = 13); on the contrary, tau phosphorylated at pThr231, pSer202 exhibited a significant decrease (*p < 0.05; **p < 0.01, non parametric Mann-Whitney test). Graphs are expressed as % Control, and means +/− SEM

To rigorously quantitate the tau burden, we performed an extensive biochemical analysis of insoluble, soluble and oligomeric tau species in brain. We monitored the tau insoluble burden (a proxy for tau aggregation) in cortex and hindbrain by low-tau ELISA (Fig. 2**,** Fig. 3). As shown in Fig. 2a, we observed a dramatic decrease of cortical insoluble tau in the P301S cohort upon treatment with scFvMC1: total tau (− 70%, **p < 0.01), pThr231 (− 70%, **p < 0.01), pSer202 (− 60%, *p < 0.05) and pSer396/404 (− 65%, **p < 0.01). The same significant reduction was confirmed in the JNPL3 (Fig. 3a) following a sustained peripheral release of scFvMC1, both as total tau (− 50%, ***p < 0.001) and phosphorylation at Thr231(− 65%,***p < 0.001), Ser202 (− 50%, ***p < 0.001) and Ser396/404 (− 60%, ****p < 0.0001).

Similar to what we observed in cortex, in the P301S mice a trend towards reduction was clearly visible in treated mice’s hindbrains (Fig. 2b): 30% reduction of insoluble total tau in hindbrain was detected although this was not significant at the *p* = 0.05 level (*p* = 0.0931); also, 40 and 50% trends to reduction were detected for pThr231 (*p* = 0.0931) and pSer202 (*p* = 0.1320), respectively. The JNPL3 treated mice’s hindbrains exhibited a significant reduction of pThr231 (− 75%, **p < 0.01) and pSer202 (− 65%, *p < 0.05), while total and tau phosphorylated at Ser396/404 were not decreased (Fig. 3b).

### Tau soluble and oligomeric species’ levels show strain differences upon treatment

**Fig. 4:**
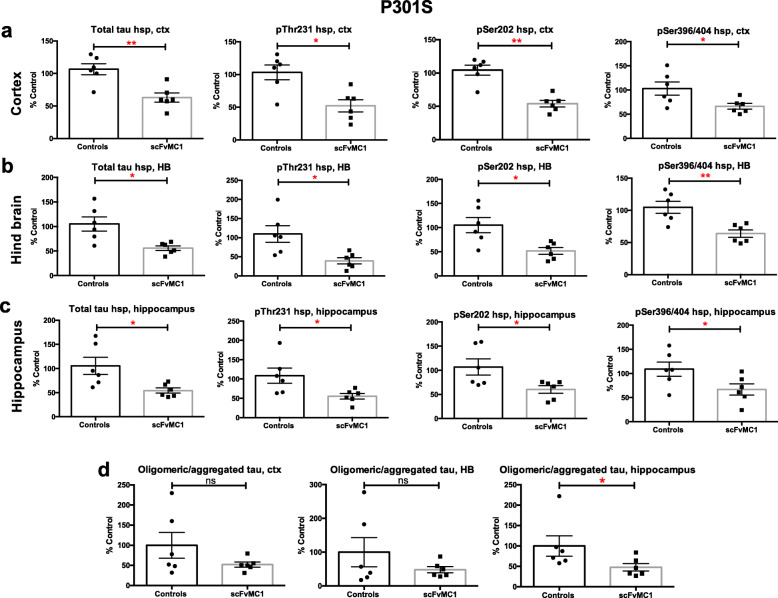
Soluble and oligomeric tau levels in P301S mice are significantly reduced. **a**, **b**, **c**, **d** P301S mice were injected at 2 month of age and sacrificed 4 months post-injection. Levels of total and phosphorylated soluble tau (pThr231, pSer202, pSer396/404) were tested on heat stable preparations (hsp) from 3 different brain regions, using low-tau ELISA: (**a**) cortex (ctx), (**b**) hindbrain (HB) and (**c**) hippocampus. In all three regions analyzed, soluble tau levels showed a significant reduction in the AAV1-CAG-scFvMC1 group (scFvMC1) (*n* = 6*)* compared to the AAV1-CAG-eGFP cohort (Controls) (n = 6) both as total and phosphorylated levels (*p < 0.05; **p < 0.01 non parametric Mann-Whitney test). (**d**) Oligomeric/aggregated tau was measured using the DA9/DA9 mono-ELISA: while a trend towards reduction was found across the cortex and the HB, the scFvMC1 treated mice (*n* = 6) exhibited a significant decrease of oligomeric tau in the hippocampus (*p = 0.0303, non parametric Mann-Whitney test) compared to the controls (n = 6). Graphs are expressed as % Control, and means +/− SEM

**Fig. 5:**
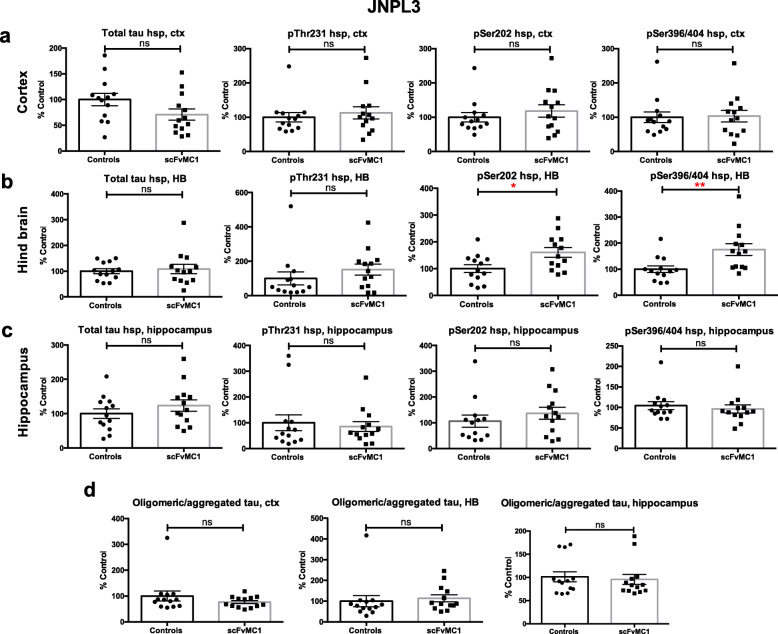
Soluble and oligomeric tau levels in JNPL3 mice. **a**, **b**, **c**, **d** JNPL3 mice were injected at 3 months of age and sacrificed 4 months post-injection. Levels of total and phosphorylated tau (pThr231, pSer202, pSer396/404) were tested on heat stable preparations (hsp) from 3 different brain regions, using low-tau ELISA: (**a**) cortex (ctx), (**b**) hindbrain (HB) and (**c**) hippocampus. Soluble tau was mostly unchanged in all brain areas in the AAV1-CAG-scFvMC1group (scFvMC1) (*n* = 13) compared to the AAV1-CAG-eGFP injected cohort (Controls) (n = 13). Two different phosphorylation sites, pSer202 (CP13 antibody, early site) and pSer396/404 (PHF1 antibody, late site) were increased in the scFvMC1 treated group (*p < 0.05, **p < 0.01, non parametric Mann-Whitney test). (**d**) Oligomeric/aggregated tau was measured using the DA9/DA9 mono-ELISA showing no modulation in any of the brain areas analyzed (cortex, ctx; hindbrain, HB; hippocampus). Controls are the AAV1-CAG-eGFP injected mice (n = 13); scFvMC1 corresponds to the AAV1-CAG-scFvMC1 group (n = 13). Graphs are expressed as % Control, and means +/− SEM

We next assessed soluble tau from cortex, hindbrain and hippocampus homogenates. Biochemical analysis of soluble tau showed differences between the two mice strains. Analysis of the P301S mice (Fig. 4) showed total and phosphorylated tau soluble species to be significantly reduced in all cerebral regions upon scFvMC1 treatment (Fig. 4a-c; 40–50% reduction**,** *p < 0.05; **p < 0.01). In contrast, in the JNPL3 mice, we did not observe any modulation of total, pThr231, pSer202 or pSer396/404 soluble tau in both cortex and hippocampus (Fig. 5a, c); surprisingly, in the hindbrain, tau phosphorylated at Ser202 and Ser396/404 was augmented in the scFvMC1injected group in JNPL3 mice (Fig. 5b).

Finally, oligomeric/aggregated tau was examined using a tau-mono-ELISA previously developed in our laboratory [65]. This assay, a functional surrogate of insoluble tau measurement, allows the detection of aggregated tau species, ranging from dimers to larger aggregates, and represents a good marker of progression towards the formation of neurofibrillary pathology in tau transgenic animal models. We routinely employ this assay for the measurement of hippocampal aggregated tau. While JNPL3 mice failed again to show any changes between controls and treated mice in all brain areas (Fig. 5d), P301S mice exhibited a significant reduction of oligomeric/aggregated tau species in the hippocampus (− 50%, *p < 0.05) and a trend towards reduction in cortex and HB (Fig. 4d), consistent with the data reported above for soluble and insoluble tau.

### Tau burden changes are not detected by immunohistochemistry

**Fig. 6:**
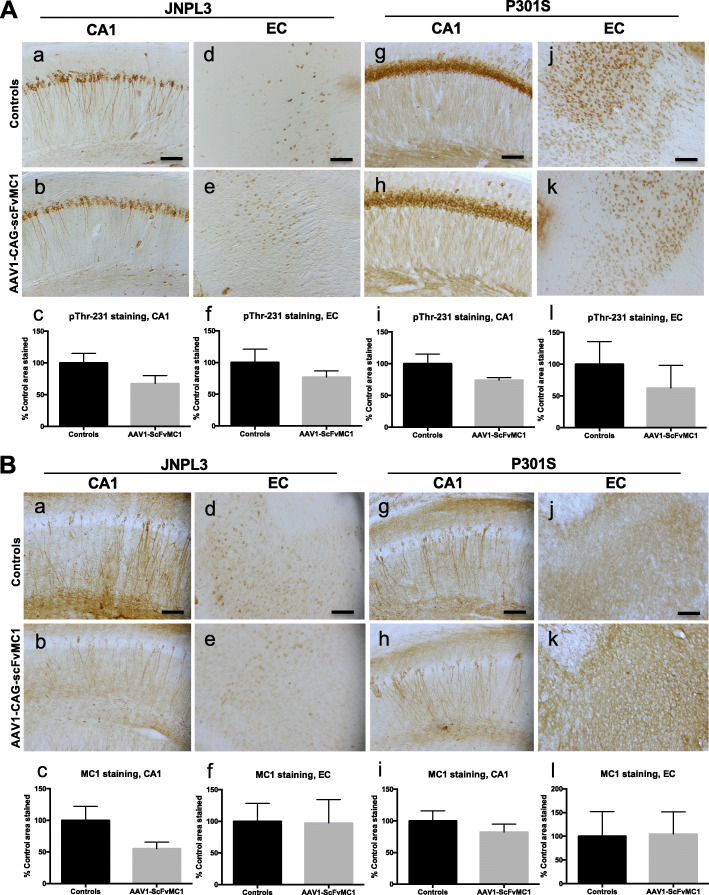
Phospho-Threonine-231 and MC1-tau immunoreactivity in brain. Brains from P301S and JNPL3 animals were harvested and stained with either anti tau phospho-Thr231 antibody (**A**) or anti-MC1-tau (**B**). (**A**): **a, b, d, e** Representative images of CA1 hippocampal cell layer and entorhinal cortex (EC) stained with anti-pThr231. Control mice received AAV1-CAG-eGFP injection (n = 13). Treated mice were injected with AAV1-CAG-scFvMC1 (n = 13). (**A**): **c**,**f** Semi-quantification of percentage of area stained by RZ3 shows a trend of reduction in the AAV1-CAG-scFvMC1 injected group, in the CA1 region of hippocampus in the JNPL3 (**c**, *p* = 0.1128, unpaired t test with Welch’s correction); no trend to reduction is detected in the entorhinal cortex (EC) **(f**, *p* = 0.3458, unpaired t test with Welch’s correction). (**A**): **g**, **h**, **j**, **k** Representative images of the CA1 region and entorhinal cortex (EC) from P301S; pThr-231 staining was performed as above. Control mice received AAV1-CAG-eGFP injection (n = 6), treated mice were injected with AAV1-CAG-scFvMC1 (n = 6); (**A) i, l** Semi-quantification of percentage of area stained by RZ3 in P301S mice (**i**, *p* = 0.2403; **l**, *p* = 0.2251; non parametric Mann-Whitney test). (**B**): **a**, **b**, **d**, **e** Representative images of CA1 hippocampal cell layer and entorhinal cortex (EC) stained with anti-MC1 antibody. Control mice received AAV1-CAG-eGFP injection (n = 13). Treated mice were injected with AAV1-CAG-scFvMC1 (n = 13) (**c**, **f**) Semi-quantification of percentage of area stained by MC1 shows a trend of reduction in the AAV1-CAG-scFvMC1 injected group, in the CA1 region of hippocampus (**c**, *p* = 0.0996; unpaired t test with Welch’s correction); no reduction is detected in the entorhinal cortex (EC) (**f**, *p* = 0.9558; unpaired t test with Welch’s correction) in JNPL3. (**g**, **h**, **j**, **k**) Representative images of the CA1 region and the entorhinal cortex (EC) from P301S; MC1 staining was performed as above. Control mice received AAV1-CAG-eGFP injection (n = 6), treated mice were injected with AAV1-CAG-scFvMC1 (n = 6). (**i, l**) Semi-quantification of percentage of area stained by MC1 in P301S mice (**i**, *p* = 0.4127; **l**, *p* = 0.8413; non parametric Mann-Whitney). (Olympus BH-2 bright field microscope; scale bar: 100 μm). Graphs are expressed as % Control area stained, and means +/− SEM

In addition we performed an immunohistochemical analysis on JNPL3 (Fig. 6A a-f; Fig. 6B a-f) and P301S brain slices (Fig. 6A **g-l**; Fig. 6B **g-l**), by assessing tau pathology in the CA1 hippocampal pyramidal cell layer and in the entorhinal cortex (EC). Semi-quantification of tau phosphorylated at Thr231 and MC1-tau was performed on both CA1 (Fig. 6A **c**, **i**; Fig. 6B **c**, **i**) and EC (Fig. 6A **f**, **l**; Fig. 6B **f**, **l**). No significant changes in immunoreactivity were observed, except some trends towards decreased staining in the hippocampal region of JNPL3 mice (Fig. 6 A c, *p* = 0.1128; Fig. 6 B c, *p* = 0.0996). Tau phosphorylation at Ser396/404 and Ser202 (not shown) also failed to show significant reduction when comparing treated to non-treated animals.

### Microglia are not activated, but have the ability to uptake the tau-scFvMC1 immunocomplex

**Fig. 7:**
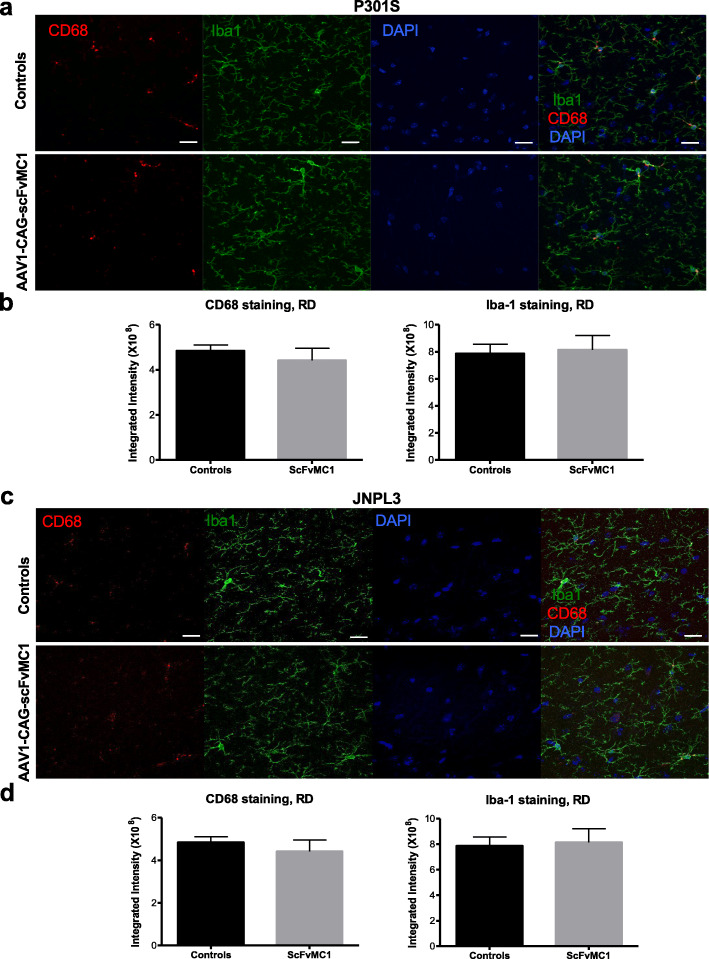
Microglia activation state does not change upon scFvMC1 treatment. For analysis of activation state, sections were immunolabeled for CD68 and Iba-1 and imaged. **a** Representative image of microglia from P301S controls (n = 6) (upper images) and AAV-scFv-MC1 treated mice (n = 6) (lower images), in the stratum radiatum: CD68 lysosomal protein (red puncta), Iba1 (green), nuclei stained with DAPI (blue). **b** Quantification of Iba-1 and CD68 showed no significant differences between controls and treated mice (*p* = 0.0931 and *p* = 0.7771 respectively, non parametric Mann-Whitney test). **c** Representative image of microglia from JNPL3 controls (n = 13) (upper images) and AAV-scFv-MC1 treated mice (n = 13) (lower images), in the stratum radiatum. **d** Quantification of Iba-1 and CD68 showed no significant differences between controls and treated mice in JNPL3 mice (*p* = 0.8857 and *p* = 0.4857 respectively, non parametric Mann-Whitney test). All images were obtained using Zeiss 880 confocal laser microscope, scale bar: 20 μm. Graphs are expressed as integrated density, and means +/− SEM

We next sought to determine whether microglia were activated in our system. Confocal microscopy was performed on brains from P301S (Fig. 7a, b) and JNPL3 (Fig. 7c, d) mice to assess the immunoreactivity of two widely studied markers for microglia activation: Iba1 (morphology/activation) and CD68 (reactive/phagocytic). We observed no difference between controls and scFvMC1 treated mice and confirmed these data by semi-quantitative analysis in the stratum radiatum of both strains. Cortex and HB were imaged and quantified displaying similar results (not shown).

**Fig. 8:**
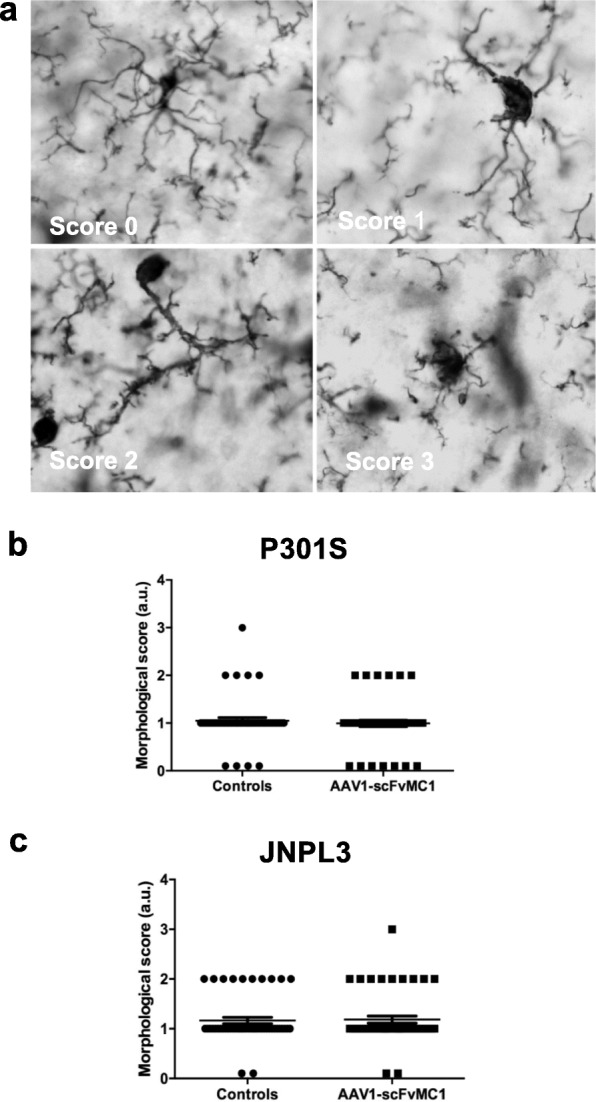
Microglia morphology is unchanged upon treatment. Microglia processes morphology was assessed in both P301S and JNPL3 animals. **a** Representative images of Iba-1 positive microglia (stratum radiatum of the CA1 subfield of the hippocampus): the processes morphology was scored as 0 (> 15 thin processes with multiple branches), 1 (5–15 thick processes with branches), 2 (1–5 thick processes with few branches), 3 (no clear processes). **b** Microglia from P301S mice did not show any significant morphological changes comparing controls to treated mice. Each single point represents a single cell (*n* = 50 cells per group, *p* = 0.5671, non parametric Mann Whitney test). **c** No significant changes in microglia morphology were detected in the JNPL3 cohort (n = 50 cells per group, *p* = 0.9628, non parametric Mann Whitney test). Graphs are expressed as arbitrary unit (a.u.) and means +/− SEM. (AxioImager Z1 microscope, Zeiss; 63x oil and 0.58 μm z-steps)

To examine deeper the microglia activation state, a morphological assessment was performed (Fig. 8a-c), using a protocol previously described [66], confirming that neither P301S or JNPL3 treated mice exhibited any significant changes in microglia morphology compared to their respective controls.

**Fig. 9:**
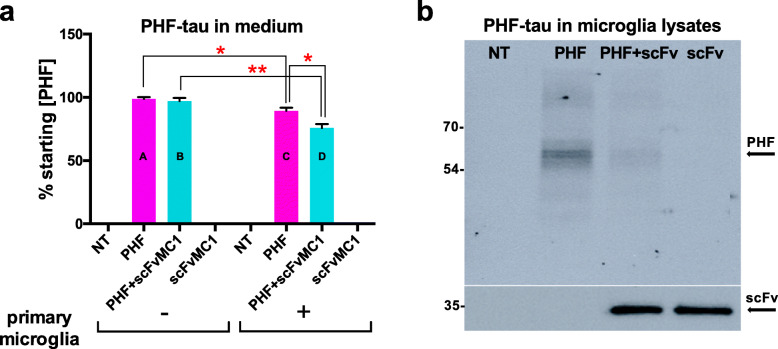
Microglia uptake phosphorylated tau in vitro*,* facilitated by scFvMC1. (**a**) Primary mouse microglia (P2 C57Bl/6 J pups) were treated in vitro for 2 h with PHF-tau +/− scFvMC1 (scFv/PHF ratio of 10/1). Total tau ELISA. Column A**:** PHF levels are expressed as % of starting PHF concentration measured after incubation on cell-free plates (− primary microglia); column B**:** amount of PHF in medium upon combination with scFvMC1, on cell-free plates (− primary microglia); column C and column D**:** PHF levels on microglia seeded plates (+ primary microglia), with or without scFvMC1. A vs C (*p < 0.05, unpaired t test with Welch’s correction); C vs D (*p = 0.0137, unpaired t test with Welch’s correction); B vs D (**p = 0.0019 unpaired t test with Welch’s correction). Data are collected from three different experiments, with treatments run in quadruplicates. Graphs are means +/− SEM. (**b**, upper panel) Representative immuno-blotting (PHF1 antibody: anti-pSer396/404) of PHF-tau in the corresponding microglia lysates; (**b**, lower panel) scFvMC1 expression verified using anti Myc-tag antibody. NT is non treated microglia; PHF is microglia treated with PHF-tau; PHF + scFv is microglia co-treated with PHF-tau and scFvMC1; scFv is microglia treated with scFvMC1

Given the peripheral injection and the lack of Fc region in the scFv structure, we did not expect an inflammatory response dependent on the microglia Fc receptor (FcR) engagement in our model, but we assumed that the altered functional state of the microglia found in neurodegenerative disorders and tau mouse models [77–80] may still facilitate microglia-mediated tau clearance upon scFv treatment. Hence, to deepen our understanding on the microglia’s role in our system, we first set out a cell-based proof of concept experiment to determine whether primary microglia could uptake the tau-scFvMC1 immunocomplex. Primary microglia were incubated for 2 h with PHF-tau (paired helical filament, prepared in our lab [69, 81]) with and without scFvMC1 (scFv/PHF ratio 10/1) (Fig. 9). In order to work in the appropriate experimental conditions, PHF-tau and PHF-tau/scFvMC1 immunocomplex were applied on plastic plates (− primary microglia) or on microglia seeded plates (+ primary microglia). Primary microglia showed an innate ability to uptake PHF from the medium (Fig. 9a**,** A vs C: 10% reduction, *p = 0.0168), which was enhanced by the presence of scFv-MC1: a 20% reduction (Fig. 9a, C vs D, *p = 0.0137) was detected when co-treating the microglia with scFvMC1/PHF compared to PHF alone. Also, in the co-treatment paradigm we observed a 25% reduction of PHF in medium (Fig. 9a, B vs D, **p = 0.0019) when working on microglia seeded plates compared to empty plates. Interestingly, intracellular PHF-tau readily disappeared, after uptake, from the microglia lysates when scFvMC1 was present (Fig. 9b, upper panel). Moreover, the levels of scFvMC1 were unchanged in microglia lysates, with and without PHF, suggesting an excess of scFv in this in vitro experiment (Fig. 9b**, lower panel**).

**Fig. 10:**
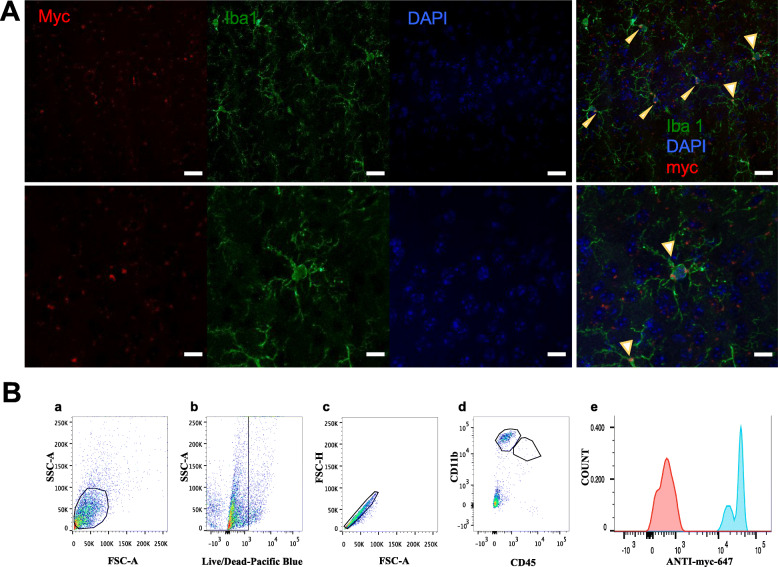
Microglia uptake scFvMC1 in vivo*.* (**A)** P301S were injected intracranially in the CA1 quadrant of the hippocampus using AAV5-GFAP-scFvMC1. Upper panels: Representative confocal images of the cortex: scFvMC1 (Myc, red) co-localizes within the microglia (Iba1, green); nuclei stained with DAPI (blue). Lower panels: higher magnification to better visualize scFvMC1 in microglia positive cells (Zeiss880 confocal laser microscope; upper panels, scale bar: 20 μm; lower panels, scale bar: 10 μm). **(B)** Flow cytometry on microglia isolated from adult P301S mice intracranially injected with AAV5-GFAP-scFvMC1 or AAV5-null (**a-c**) Gating strategy (live, singlets) for subsequent selection of microglia. (**d**) Gating strategy to isolate microglia from other monocytes. Representative plot showing microglia population: CD11b^high^ and CD45^low^; near-complete absence of macrophages: CD11b^high^ and CD45^high^. (**e**) Microglia extracted from P301S mice, treated (blue) or not (red) with scFv-MC1: upon permeabilization, anti-Myc-647 detects scFvMC1 in microglia of treated mice (blue)

Since we were not able to directly visualize scFvMC1 in the brain upon IM injection of the vectorized antibody, to further validate our in vitro data we have utilized adult P301S mice injected intracranially with AAV5-GFAP-scFvMC1, using the same strategy applied in our previous study [43]. Confocal microscopy was performed to co-localize tau and scFvMC1 in microglia confirming that both phospho-tau (pThr231) and scFvMC1 (Myc-555) co-localize in Iba1 positive microglia (Supplementary Fig. 2). Using the same experimental model, we show by confocal microscopy (Fig. 10 A) that scFvMC1 is detected in Iba1 positive microglia from treated animals, confirming a role of this cellular population in the uptake and clearance of pathological tau. Finally, adult P301S intracranially injected with AAV5-GFAP-scFvMC1 were employed to isolate microglia [70, 77, 82, 83] from whole brain. Following microglia sorting with flow cytometry (CD11b^high^ CD45^low^) (Fig. 10 B, a-d), cells were permeabilized to detect scFvMC1 inside the microglia (anti-Myc-647, blue) in the AAV5-scFvMC1 injected mice; no anti-Myc-647 was detected in the AAV-null injected animals (red) (Fig. 10 B, e).

Overall, our in vitro and in vivo data indicate that, despite the lack of Fc effector function, microglia have the ability to uptake tau and scFvMC1.

### Antibodies directed to the scFvMC1 are detected in the JNPL3 mice serum

A major concern about the long-term use of antibodies as a treatment is the generation of neutralizing antibodies (NAB), which would compromise the therapeutic effect. We have investigated whether expression of scFv gene in the body would trigger the production of antibodies directed against scFv. Upon sacrifice, serum was collected and processed to test for the presence of scFvMC1 in the circulation. While we failed to directly detect scFvMC1 in serum, we were able to detect antibodies directed to scFvMC1 in the JNPL3 treated cohort (Supplementary Fig. 3b), similar to what observed in our previous study upon intracranial injection of AAV5-scFvMC1 [43]. Contrarily, the treated P301S mice did not show any detectable anti-scFvMC1 in serum (Supplementary Fig. 3a).

Our serological assessment was completed by determining the tau levels in the circulation, to investigate the ability of scFvMC1 to export tau from the brain parenchyma to the periphery [84]. As shown in Supplementary Fig. 3c, d tau levels did not change upon treatment in both mice models.

## Discussion

The use of antibody fragments has emerged as a promising approach to target both Aβ and tau pathology in Alzheimer’s disease [32–34, 39, 40, 42–45, 85]. We have previously reported that intracranial administration of the vectorized anti-tau scFvMC1 was able to reduce different tau species in the JNPL3 transgenic animal model [43]. This study has set the basis for the development of a novel therapeutic approach aimed at advancing peripheral administration of vectorized anti-tau scFv: in the present work, we show that IM injection of anti-tau scFv antibodies has potential for the treatment of tauopathies.

Few studies [35, 45] have previously demonstrated that intramuscular delivery of an anti-Aβ scFv gene in an AD mouse model reduced amyloid deposits and ameliorated its learning and memory deficits without inducing discernible inflammation. In line with the current strategies, we have utilized AAV1 to express scFvMC1 in the striated muscle, to generate a peripheral niche and achieve long term production of the fragment antibody. We have extended our investigation to two different models of tauopathy, reaching significant reduction of insoluble and soluble tau species, total and phosphorylated, with regional and mouse strain differences. The following considerations are in order.

Detecting scFvMC1 in the brain parenchyma using antibodies against the Myc or His tags (see construct design in [43]) has proven challenging when the scFv is produced in the periphery [35, 45], as in the current work. Contrarily, in our previous publication, where the scFv was expressed directly in the brain, we were able to detect and track the diffusion of the scFv by both IHC and western blot in the 3 brain regions analyzed (hippocampus, cortex, hindbrain) [43]. We thus hypothesized that the local brain concentration of the peripherally generated scFv would not be enough for detection. Using this systemic approach we also failed to detect scFv in serum: it is plausible that, together with the detection limits of the assay employed, a certain amount of recombinant antibody might get misfolded and degraded, explaining why we can’t visualize scFv in serum. When we conjugated the scFvMC1 to an IR dye and injected 100 μg of it by IV route (less than half the dose normally injected in anti-tau passive immunotherapy experiments in mice, i.e. 10 mg/kg), we were able to detect the compound in the brain, and similarly for the native MC1 antibody. Importantly, the ratio between the blood and brain parenchyma concentrations are in line with what seen by others [72, 74, 86, 87] for both the native and scFv antibody. Hence, these data suggest that our scFv is able to cross the BBB and reach its target, justifying its use in a systemic delivery paradigm. Consistent with this proof-of-concept experiment, the efficacy data show that peripherally generated scFvMC1 can reach the brain parenchyma to modulate tau levels, with no peripheral leakage of the transgene expression, conferring to this approach a relevant translational advantage.

Previous studies from our laboratory and others [14, 16] have investigated the effect of tau passive immunotherapy using peripherally injected native MC1, in both JNPL3 and P301S mice. Comparing the present study to the previous ones is not trivial, given the different nature of the antibodies (full antibody vs scFv), the variability of the mice strains and the delivery routes employed. One important finding in the present work is the highly significant decrease of insoluble total and phosphorylated tau in cortex, with similar response in P301S and JNPL3 mice. P301S soluble tau was also reduced across the brain, while oligomeric/aggregated tau was reduced mostly in the hippocampus. On the contrary, we did not observe any reduction of soluble and oligomeric species in the JNPL3 mice, and detected instead an increase of 2 phospho-tau epitopes in the hindbrain: we have no clear explanation at this point for the increase of soluble pSer202 (early p-tau epitope in AD) and pSer396/404 (late p-tau epitope in AD) tau in the JNPL3 HB, which is opposite to the other findings. Semi-quantitative analysis of RZ3 and MC1 immunohistochemistry staining on brain slices did not confirm the reduction of soluble tau detected biochemically by ELISA, in the P301S model, which is more sensitive to changes in soluble and insoluble tau. Taken together, these data point to regional and qualitative (soluble vs insoluble) differences in tau clearance in both mice models.

Indeed, mice strains may vary in the quantitative and topographic expression of the tau transgene (by virtue of promoters and copy numbers), rate of deposition of tau pathology and tau phosphorylation, inflammation/reactive changes and BBB permeability to the scFv. As previously reported, in contrast to the JNPL3 model, the P301S mice accumulate detectable levels of hyperphosphorylated tau species (64-kDa) in soluble as well as insoluble fractions [16, 63]. The precise reason for this difference between the pathological tau accumulation in the two models is unclear but may explain some discrepancy in the effect of anti-tau immunotherapy.

The use of antibodies to tackle tau pathology is fascinating, but may carry consequences in terms of eliciting an inflammatory response, by activation of immune cells via Fc receptor. In our system, the absence of the Fc region should prevent such occurrence. Indeed, we have not found any signs of microglia activation, nor we have found signs of peripheral tissue damage from the AAV-scFvMC1 transduction, or increased pro-inflammatory cytokines levels in serum. Hence, so far, our strategy appears to be safe. However, the host immune system could pose some limitations, such as cellular immune responses to AAV vector and/or transgene. In this respect, the AAV field is moving into developing strategies to overcome immunogenicity produced by re-administration [88]. In our study, we have detected antibodies directed to scFvMC1 in the JNPL3 cohort, which did not prevent efficacy, in line with what reported earlier [43].

Depending on the administration route, the dose, and/or the AAV serotype, gene transfer can result in a deleterious immune response [89, 90] or tolerance [61, 91, 92]. Two studies have shown that AAV1 IM injection can lead to regulatory T cells (Tregs) infiltration in injected muscles in AATD (Alpha-1 Antitrypsin Deficiency) and LPLD (Lipoprotein Lipase Deficiency) patients [61, 91]. Gernoux et al. [92] have recently correlated results obtained in monkeys to those obtained with patients and further demonstrated that tolerance and persisting transgene expression after AAV1 gene transfer in muscle is mediated by Tregs and exhausted T cells. These findings support the IM approach proposed in the present study and its translational potential for humans.

One last mechanistic aspect was investigated: the role of microglia in clearing tau after scFvMC1 administration. Luo et al. [93] have reported that murine microglia rapidly internalize and degrade hyperphosphorylated pathological tau isolated from AD brain tissue in a time-dependent manner in vitro, showing that the anti-tau monoclonal antibody MC1 had the ability to enhances microglia-mediated tau degradation in an Fc-dependent manner. Despite the lack of Fc in our scFvMC1, we wanted to investigate this mechanistic aspect, assuming that the microglia could still participate in the removal of the immunocomplex in our model. Thus, we took both an in vitro and in vivo approach to determine if microglia are able to uptake PHF (hyperphosphorylated aggregated tau) with or without the help of scFv. We found that in vitro, cultured microglia were able to uptake PHF-tau (confirming the previous study [93]) and scFvMC1 had the ability to enhances this process; furthermore, scFvMC1 accelerated the degradation of PHF tau in these cells. In vivo, we have been able to co-localize tau (anti-pThr231), scFvMC1 (anti-Myc) and microglia cells (anti-Iba1) after intracranial injection of AAV-scFvMC1. It is plausible that multiple mechanisms contribute to the clearance of tau upon scFv immunotherapy and that microglia uptake is one of the routes the brain uses to remove pathological tau. Future experiments will investigate whether microglia or other CNS or peripheral cell types participate in the uptake of scFvMC1 and tau using receptors other than the FcR, i.e. toll-like receptors (TLRs), C-X3-C motif chemokine receptor 1 (CX3CR1), pattern-recognition receptors (PRRs), scavenger receptors, and complement protein C1q receptor (C1q-R).

## Conclusions

To our knowledge, this work provides the first description of vectorized anti-tau scFv exerting an effect in the brain upon intramuscular injection. More studies are planned to investigate whether the highly significant reduction of insoluble tau gained in both animal models will also ameliorate their behavioral phenotype. Also, from a clinical perspective, particularly attractive would be the ability to not only express the scFv but to switch it on or off at will, adding a layer of exogenous control to improve safety. Among the currently available inducer/repressor systems permitting control over gene expression, the tetracycline (Tet)-dependent system is by far the most advanced and most widely used, and has been already tested in association with AAV1 and locoregional muscle gene transfer in non-human primates showing no humoral or cellular responses against the transgene [94]: we will certainly explore this avenue in our system.

In addition, our work opens the field to future studies reaching beyond scFv, to test engineered antibodies with multiple specificities and targets in the brain, using a simple and translatable approach.

Overall, given the limitations linked to conventional immunotherapy in neurodegenerative diseases, this work demonstrates the efficacy and the advantages of using intramuscular injection of vectorized scFv to target tau, and its relevant translational features, suggesting potential applications to other brain proteinopathies.

## Supplementary information

**Additional file 1 Supplementary figure 1:** Inflammatory status in peripheral organs. NF-kB immunoreactivity, marker of activated proinflammatory pathways, was evaluated on kidney, liver, tibialis muscle and heart: representative images of each organs do not show differences between controls and AAV1-scFvMC1 treated mice (Controls = AAV1-CAG-eGFP; Bright field microscope, scale bar: 300 μm).

**Additional file 2 Supplementary figure 2.** Phospho-tau and scFvMC1 co-localize in microglia. Representative confocal image of the stratum radiatum from P301S mice injected with AAV5-GFAP-scFvMC1. Astrocytes actively express scFv-MC1 (Myc-red); Iba1 positive microglia (green) shows co-localization of scFvMC1 (myc-red) and pTau (pThr231; blue): merge purple (white arrows). Zeiss880 confocal laser microscope: merge image is 2x crop of 40X magnification; scale bar: 20 μm.

**Additional file 3 Supplementary figure 3** Anti-scFvMC1and tau in serum. **(a, b**) Antibodies anti-scFvMC1 were assessed using a specific immune sorbent assay: JNPL3 mice receiving the scFvMC1 exhibited a significant increase of anti-scFvMC1 in serum (***p = 0.0005, non parametric Mann-Whitney test) compared to the controls; no anti-scFvMC1 were detected in the P301S animals; (**c**, **d**) total tau levels were measured in serum at the end of the 4 month treatment: no significant changes were identified in both transgenic models, P301S and JNPL3. Graphs are expressed as Arbitrary Units (**a**, **b**) or % Control (**c**, **d**), and means +/− SEM.

## Data Availability

The datasets used and/or analyzed during the current study are available from the corresponding authors upon reasonable request.

## Abbreviations

Ab: Antibody
Aβ: Amyloid-beta
AAV: Adeno-associated viral vector
AD: Alzheimer’s disease
BBB: Blood-brain barrier
CNS: Central nervous system
Ctx: Cortex
EC: Entorhinal cortex
HB: Hind brain
Hippo: Hippocampus
IM: Intramuscular
IV: Intravenous
LV: Lentiviral vector
mAbs: Monoclonal antibodies
PHF: Paired helical filaments
RD: Stratum radiatum
scFv: Single chain variable fragment
scFvMC1: Single chain variable fragment MC1
Tregs: Regulatory T cells
V_H_: Variable heavy chain
V_L_: Variable light chain
