# Supplementary figures and images for: Intramuscular injection of vectorized-scFvMC1 reduces pathological tau in two different tau transgenic models
Source: Acta Neuropathol Commun. 2020 Aug 6;8:126. doi: 10.1186/s40478-020-01003-7 (PMC7409655; doi:10.1186/s40478-020-01003-7)

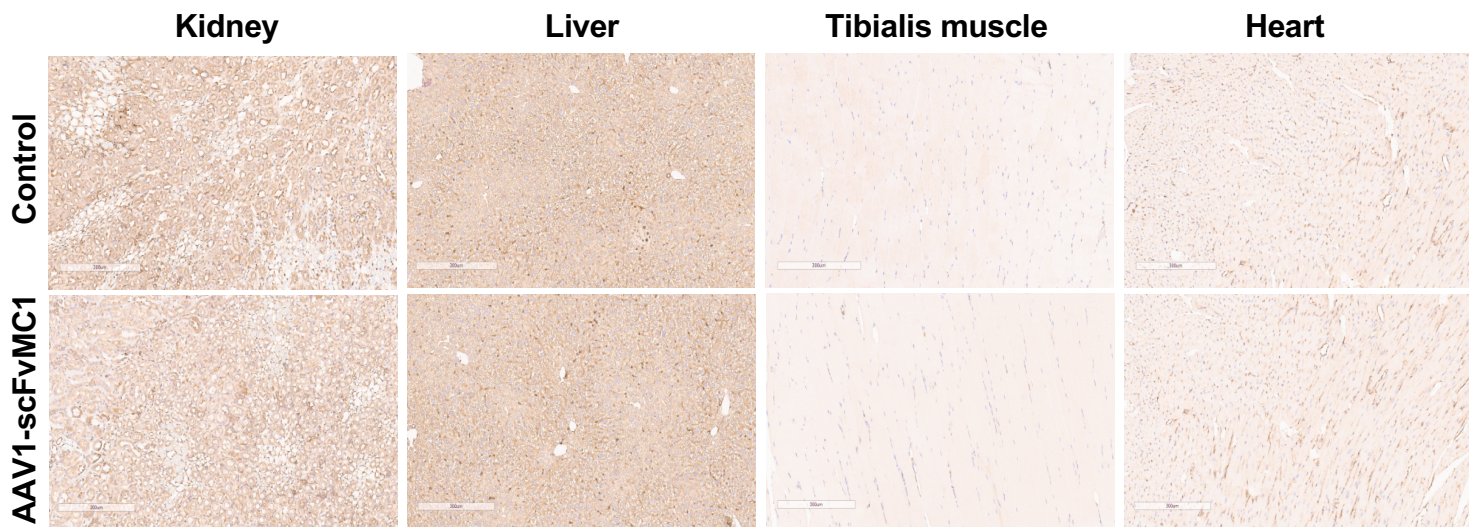

Supplement: Supplementary file 1 — Additional file 1 Supplementary figure 1: Inflammatory status in peripheral organs. NF-kB immunoreactivity, marker of activated proinflammatory pathways, was evaluated on kidney, liver, tibialis muscle and heart: representative images of each organs do not show differences between controls and AAV1-scFvMC1 treated mice (Controls = AAV1-CAG-eGFP; Bright field microscope, scale bar: 300 μm). [file 40478_2020_1003_MOESM1_ESM.pdf]

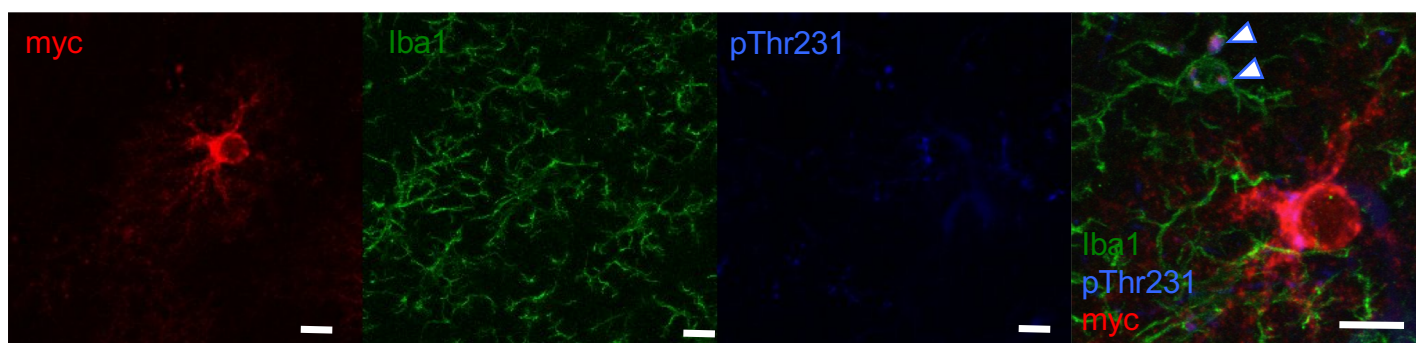

Supplement: Supplementary file 2 — Additional file 2 Supplementary figure 2. Phospho-tau and scFvMC1 co-localize in microglia. Representative confocal image of the stratum radiatum from P301S mice injected with AAV5-GFAP-scFvMC1. Astrocytes actively express scFv-MC1 (Myc-red); Iba1 positive microglia (green) shows co-localization of scFvMC1 (myc-red) and pTau (pThr231; blue): merge purple (white arrows). Zeiss880 confocal laser microscope: merge image is 2x crop of 40X magnification; scale bar: 20 μm. [file 40478_2020_1003_MOESM2_ESM.pdf]
