# Supplementary material for: Intramuscular injection of vectorized-scFvMC1 reduces pathological tau in two different tau transgenic models
Source: Acta Neuropathol Commun. 2020 Aug 6;8:126. doi: 10.1186/s40478-020-01003-7 (PMC7409655; doi:10.1186/s40478-020-01003-7)
Supplement: Supplementary file 3 — Additional file 3 Supplementary figure 3 Anti-scFvMC1and tau in serum. (a, b) Antibodies anti-scFvMC1 were assessed using a specific immune sorbent assay: JNPL3 mice receiving the scFvMC1 exhibited a significant increase of anti-scFvMC1 in serum (***p = 0.0005, non parametric Mann-Whitney test) compared to the controls; no anti-scFvMC1 were detected in the P301S animals; (c, d) total tau levels were measured in serum at the end of the 4 month treatment: no significant changes were identified in both transgenic models, P301S and JNPL3. Graphs are expressed as Arbitrary Units (a, b) or % Control (c, d), and means +/− SEM. [file 40478_2020_1003_MOESM3_ESM.pdf]

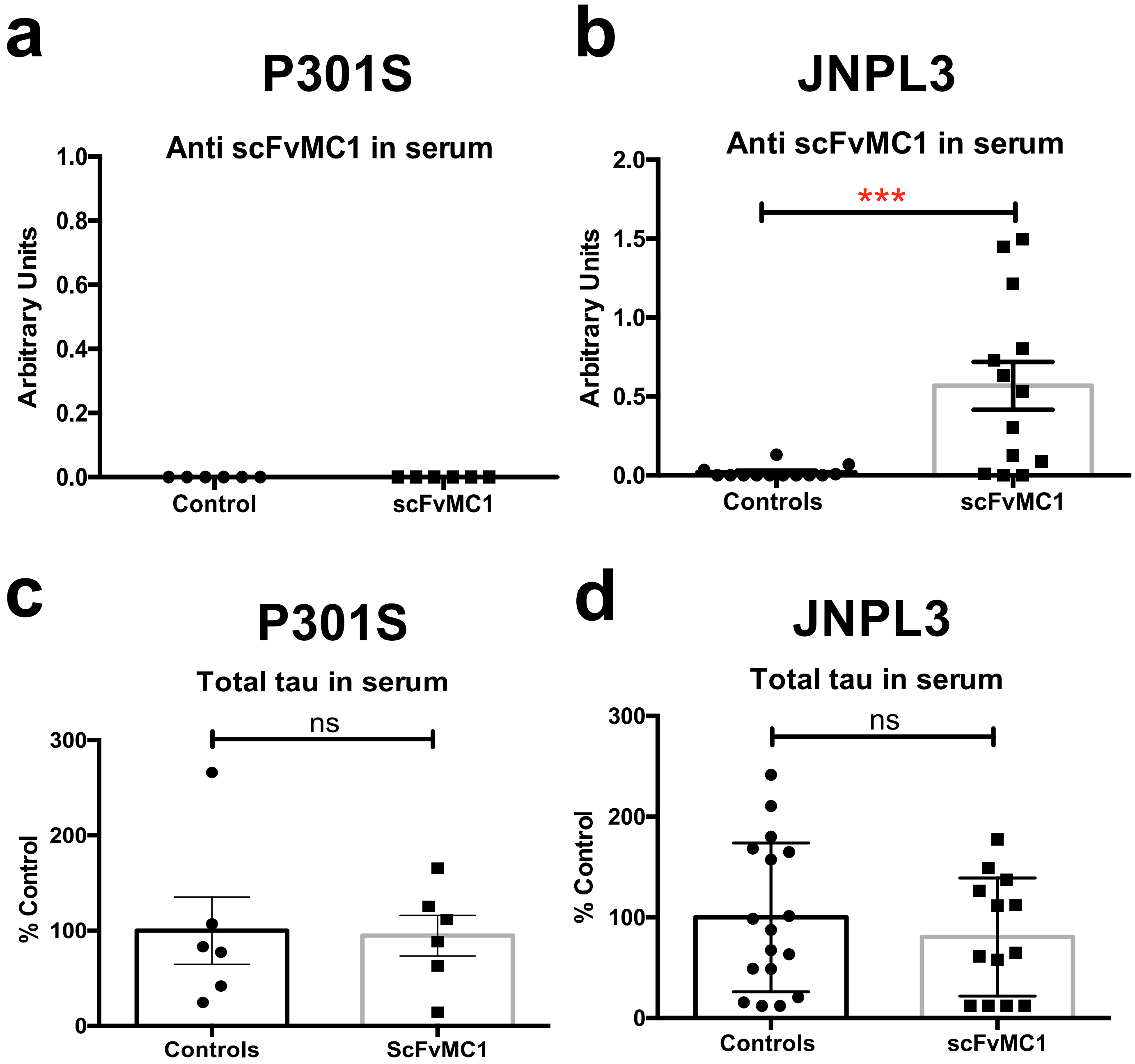

a
P301S
Anti scFvMC1 in serum
Arbitrary Units
1.0
0.8
0.6
0.4
0.2
0.0
Control
scFvMC1
b
JNPL3
Anti scFvMC1 in serum
Arbitrary Units
2.0
1.5
1.0
0.5
0.0
***
Controls
scFvMC1
c
P301S
Total tau in serum
% Control
300
200
100
0
ns
Controls
ScFvMC1
d
JNPL3
Total tau in serum
% Control
300
200
100
0
ns
Controls
scFvMC1
